# Growth and inactivation of *Salmonella enterica* and *Listeria monocytogenes* in broth and validation in ground pork meat during simulated home storage abusive temperature and home pan-frying

**DOI:** 10.3389/fmicb.2015.01161

**Published:** 2015-10-27

**Authors:** Xiang Wang, Evy Lahou, Elien De Boeck, Frank Devlieghere, Annemie Geeraerd, Mieke Uyttendaele

**Affiliations:** ^1^Laboratory of Food Microbiology and Food Preservation, Department of Food Safety and Food Quality, Faculty of Bioscience Engineering, Ghent UniversityGhent, Belgium; ^2^MeBioS, Department of Biosystems (BIOSYST), Faculty of Bioscience EngineeringKU Leuven, Leuven, Belgium

**Keywords:** *Salmonella enterica*, *Listeria monocytogenes*, ground pork meat, growth kinetics, thermal inactivation, home pan-frying

## Abstract

Ground pork meat with natural microbiota and inoculated with low initial densities (1–10 or 10–100 CFU/g) of *Salmonella enterica* or *Listeria monocytogenes* was stored under abusive temperature at 10°C and thermally treated by a simulated home pan-frying procedure. The growth and inactivation characteristics were also evaluated in broth. In ground pork meat, the population of *S. enterica* increased by less than one log after 12-days of storage at 10°C, whereas *L. monocytogenes* increased by 2.3 to 2.8 log units. No unusual intrinsic heat resistance of the pathogens was noted when tested in broth at 60°C although shoulders were observed on the inactivation curves of *L. monocytogenes*. After growth of *S. enterica* and *L. monocytogenes* at 10°C for 5 days to levels of 1.95 log CFU/g and 3.10 log CFU/g, respectively, in ground pork meat, their inactivation in the burger subjected to a simulated home pan-frying was studied. After thermal treatment *S. enterica* was undetectable but *L. monocytogenes* was recovered in three out of six of the 25 g burger samples. Overall, the present study shows that data on growth and inactivation of broths are indicative but may underestimate as well as overestimate behavior of pathogens and thus need confirmation in food matrix conditions to assess food safety in reasonably foreseen abusive conditions of storage and usual home pan-frying of meat burgers in Belgium.

## Introduction

*Salmonella enterica* and *Listeria monocytogenes* are two of the most important foodborne pathogens; they are known to occur in raw meat, and are associated with foodborne outbreaks (Rose et al., 2002; EFSA and ECDC, 2015). Consuming contaminated raw or undercooked meat is believed to be one of the important vehicles of foodborne infection. The presence of these pathogens in meat can present a serious food safety threat. According to the strong-evidence foodborne outbreaks in Europe, up to 38.5% cases happened at households/domestic kitchens (EFSA and ECDC, 2015). Adequate refrigeration and thorough cooking are two points of attention to ensure microbiological safety of meat toward the end of the food chain.

Ground meat is a potentially hazardous type of fresh meat, it is particularly susceptible to bacterial contamination throughout its mass, and therefore, more likely to contain foodborne pathogens (Lianou and Koutsoumanis, 2009; Schlisselberg et al., 2013). Both retailers and consumers use low storage temperatures to minimize growth of spoilage and pathogenic microorganisms. However, *L. monocytogenes* can survive or even grow at low temperatures; *S. enterica* can grow when the storage temperatures are abused. Predictive models can be used to estimate the growth potential of microorganisms in the food chain. A number of models and software have been developed to predict the effects of temperature, pH or water activity on the growth of pathogens in ground meat (Mbandi and Shelef, 2001; Ingham et al., 2007; Pin et al., 2011; Velugoti et al., 2011). A limitation of these models is that they are based on the collection of data in sterile ground meat. Studies have demonstrated that the effects of competing microbiota on the growth of pathogens cannot be neglected (Zaher and Fujikawa, 2011; Møller et al., 2013). Turning our attention to the growth in ground pork meat, studies concerning the effect of natural microbiota on growth of pathogens have been performed by Ingham et al. (2007) and Møller et al. (2013) where ground pork was inoculated with relatively high levels of pathogens (3–5 log CFU/g). However, the actual initial contamination level of *S. enterica* and *L. monocytogenes* in ground pork is usually low (<10–100 CFU/g) (Ghafir et al., 2005; Thevenot et al., 2006). Studies on chicken meat and fresh cut salads have indicated that the pathogens’ initial densities had effects on their growth in the presence of natural microbiota (Oscar, 2007; Manios et al., 2013), and we expect a similar effect in ground pork meat. The growth of *S. enterica* and *L. monocytogenes* in ground pork meat with realistic levels of natural microbiota and low levels of inoculated pathogens is, as far as the authors are aware of, not available in literature or in the Combase Browser^1^.

Home-cooking practice is an important and effective way to eliminate pathogens in meat. So far, thermal treatment remains the principal method of microbial inactivation for consumers at home (Álvarez-Ordóñez et al., 2008). It is recommended that ground pork or beef must be cooked to an internal temperature of 71 or 70°C for 2 min or its equivalent (Advisory Committee on the Microbiological Safety of Food [ACMSF], 1995; FDA, 2011b). However, most of the European consumers check the meat doneness visually, rather than using a thermometer (Bearth et al., 2014). Information used to establish cooking recommendations has largely been derived from *D* values in laboratory experiments (International Commission on Microbiological Specifications of Foods [ICMSF], 2005). Since the late 1990s, a number of studies have evaluated the heat resistance of *S. enterica* and *L. monocytogenes* in buffers or broth (Juneja et al., 2001; Sorqvist, 2003; Miller et al., 2009), and in meat and meat products (Juneja et al., 2001; Murphy et al., 2006; Halder et al., 2010; Vasan et al., 2014), but data collected using actual consumer-based handling and cooking processes are comparatively scarce. Thermal inactivation studies in the laboratory are usually performed at isothermal conditions, yet the cooking processes consumers use at home are generally non-isothermal: burgers are usually thermally treated for several minutes on each side in a frying pan in hot butter before being served for consumption. Furthermore, microorganisms in ground meat are immobilized and constrained to grow as colonies rather than planktonically, which may also have an effect on the observed thermal inactivation profiles. So far, no study has focused on the inactivation of foodborne pathogens, with the latter being previously allowed to grow in ground meat, providing, thus, the rationale for setting up and conducting the present study.

For assessing the food safety it is needed to estimate the growth and survival of pathogens in meat under reasonable foreseen conditions of pathogens’ contamination level as well as storage conditions and subsequent thermal treatment prior to consumption. The average temperature of the fridge of Belgian households is 6.7°C and as much 10.8% (*n* = 3001) was even at temperatures larger than 10°C (De Vriese et al., 2005). Therefore, we conducted a systematic study to assess the behavior of *S. enterica* and *L. monocytogenes* in ground pork meat under 10°C refrigerator storage and subsequent consumer-based pan frying with, as usually practiced in Belgium, visual assessment of doneness. Ground pork with natural microbiota and inoculated with a low initial density (1–10 or 10–100 CFU/g) of *S. enterica* and *L. monocytogenes* was used to mimic naturally contaminated burgers. Meanwhile, for comparativeness, the growth and inactivation of these pathogens were also evaluated in brain heart infusion (BHI) broth. The study will help to reduce the uncertainties in assessing the food safety threat of *S. enterica* and *L. monocytogenes* in ground pork meat. It will also permit to validate the applicability of the estimations derived from microbial growth and inactivation models often established in broth media and provide quantitative information on the behavior of *S. enterica* and *L. monocytogenes* in ground pork during reasonably foreseen home storage conditions and cooking practices.

## Material and Methods

### Bacterial Strains and Culture Conditions

The following strains of *S. enterica* and *L. monocytogenes* were used for the growth and thermal inactivation test. Of *S. enterica*, three food-isolated strains selected were *Salmonella* Derby LFMFP 872 (pork isolate), *Salmonella* Enteritidis LFMFP 875 (poultry isolate) and *Salmonella* Typhimurium LFMFP 877 (poultry isolate). Three *L. monocytogenes* strains (LFMFP 392, serotype 4b, liver pate isolate; LFMFP 421, serotype 4b, clinical isolate, and LFMFP 491, serotype 1/2b, tuna isolate) were used. All stock cultures were kept at –75°C in Tryptone Soy Broth (TSB, Oxoid, Basingstoke, England), supplemented with 0.6% yeast extract (YE, Oxoid) and 15% glycerol (Prolabo, Heverlee, Belgium). Working stocks were stored refrigerated at 4°C on Tryptone Soy Agar (TSA, Oxoid) slants and were renewed monthly. Working cultures were activated by transferring a loopful from the slants into BHI (Oxoid) and incubated at 37°C for 18 to 24 h. The working cultures were prepared by transferring 0.1 ml of each culture into 10 ml of BHI and incubated at 37°C for 24 h. Immediately before inoculation, a cocktail containing three strains of *S. enterica* or *L. monocytogenes* was prepared individually by mixing approximately equal population of each strain and serially diluted in Peptone Physiological Salt Solution (PPS, containing 1 g/l neutralized bacteriological peptone and 8.5 g/l NaCl).

### Growth Studies

#### Growth Studies in Broth

The growth curves of *S. enterica* and *L. monocytogenes* in broth at 10°C were determined in BHI. One milliliter of each pathogen cocktail dilution was inoculated into a 250-ml blue-cap bottle containing 99 ml of BHI to yield an initial dose of 1-10 (10^-7^ dilution) and 10–100 (10^-6^ dilution) CFU/ml. The broth was equilibrated overnight in the refrigerator to 10°C before inoculation. The incubation period was 24 days for *S. enterica* and 10 days *for L. monocytogenes*. At regular time intervals, aliquots (1 ml) of the culture were taken and serially diluted in PPS followed by plating on duplicated plates. The *S. enterica* and *L. monocytogenes* populations were determined by plating on Xylose Lysine Deoxycholate (XLD, Oxoid) and Listeria Ottaviani and Agosti (ALOA, Biolife, Milano, Italy), respectively. Bacterial colonies were enumerated after incubation of the plates at 37°C for 24 and 48 h for *S. enterica* and *L. monocytogenes*, respectively.

#### Growth Studies in Ground Pork Meat

Ground pork meat was purchased at a local store and analyzed for the presence of *S. enterica* and *L. monocytogenes*, and was found to be absent in 25 g of meat samples (see below). The analysis of characteristics of the meat was performed as described by Lahou et al. (2015). It indicated that the ground pork contains about 8.1% fat and 1.5% sodium salt. The measured pH and water activity were 5.6 and 0.98, respectively. The meat was divided into portions (9.9 g) and aseptically transferred into a stomacher bag for growth studies. A diluted culture (0.1 ml) of the cocktail of *S. enterica* or *L. monocytogenes* was inoculated individually. The initial pathogen density aimed for was 1–10 or 10–100 CFU/g, which is similar to the level expected in naturally contaminated meat. The negative control samples were inoculated with 0.1 ml PPS. After the inoculum was added, the bags were hand mixed for 30 s, stomached for 2 min, compressed into a thin, uniform layer, loosely heat sealed, and then stored in a 10°C refrigerator. At selected times of incubation samples were added with 90 ml of PPS and were thoroughly homogenized in a stomacher (Lab Blender 400, Seward Laboratory, London, UK). Each sample was then serially 10-fold diluted with PPS for determination of bacterial density. The enumeration of the total plate count (TPC) in Plate Count Agar (PCA, Oxoid) was derived from ISO 6222 (5 days incubation at 22°C). Presumptive lactic acid bacteria (LAB) count was determined on Man Rogosa Sharp Agar (MRSA, Oxoid) with an overlay according to ISO 15214 (3 days incubation of MRS at 30°C) and the enumeration of coliforms was performed using Violet Red Bile Lactose (VRBL, Oxoid) Agar overlaid with the same medium according to ISO 4832 (24 h incubation of VRBL at 37°C). *S. enterica* and *L. monocytogenes* was, respectively, plated on XLD and ALOA plates. Suspected *S. enterica* colonies were further confirmed using Crystal E/NF ID (BD Benelux N. V, Erembodegem, Belgium).

### Thermal Treatments

#### Thermal Treatment in Broth

**FIGURE 1:**
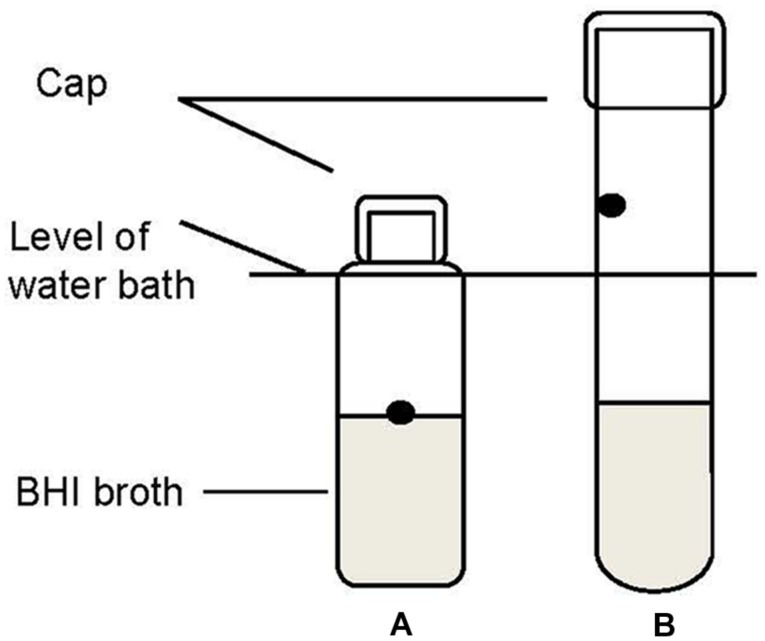
**Schematic diagram of method I (A, reference method) and method II (B, alternative method) used to assess the heat resistance of pathogen strains in the water bath.** The dots are the spots where cultures were injected.

Two methods were compared to evaluate whether different test methods used to measure thermal inactivation would influence the results. The schematic diagram of the two methods is shown in **Figure 1**. In Method I (**Figure 1A**), a 0.1 ml portion of the stationary phase culture was added directly into 9.9 ml BHI in test tubes (125 mm × 15 mm), resulting in an initial population of approximately 7.0 log CFU/ml. This method is termed the reference method. Method II is referred to as an alternative method. In method II 1-ml portions of culture were inoculated to 9 ml of BHI along the inner wall of the thin-walled test tube (160 mm × 15 mm) (**Figure 1B**). In both methods the test tubes were submerged in a water bath (Memmert, WB 10, Germany) preheated to the target inactivation temperature of 60 ± 0.1°C. The temperature of the broth was monitored in a test tube throughout the duration of the thermal treatment with Testo 177-T4 temperature data logger (Testo AG, Lenzkirch, Germany). After the treatment, all the tubes were transferred to an ice water bath within 30 min before plating on XLD or ALOA plates for survivors.

Heat resistance of all the bacterial strains was compared in standard BHI broth (pH 7.3, 0.5% NaCl) and in BHI adjusted to pH 5.6 with lactic acid and NaCl 1.5% (w/w) as the intrinsic conditions in the ground pork meat. The added volume of lactic acid did not significantly affect the volume of the media. The stationary phase cultures of each tested strain were separately diluted with the challenge media (standard or adjusted BHI) to around 6 log CFU/ml. For the heat resistance test, 1-ml portions of the diluted culture were thermal treated as described in method II previously.

#### Thermal Treatment in Pork Meat Burgers upon Simulated Home Pan-frying

One milliliter strain mixture dilution of *S. enterica* or *L. monocytogenes* was individually inoculated into 99 g portions of ground pork in a stomacher bag for an initial dose of 10–100 CFU/g. The stomacher bags were massaged as described previously. Burgers (8.5 cm by 1.5 cm) were prepared in sterile Petri dish. Individual burgers were placed in stomacher bags, heat sealed, stored at 10°C for 5 days, and subjected to microbial analysis and simulated home pan-frying.

The inoculated pork burgers were baked in a frying pan of TEFAL S.A.S^^®^^ with a diameter of 24 cm on an electrical heating plate (SCHOTT^^®^^ instruments, model: SLK2, 1800 W, heated zone diameter of 20 cm). The standardized cooking procedure and time was established based on preliminary tests as to obtain a visual well-done cooked pork burger (Lahou et al., 2015). The pan was preheated at heating state 7 (the highest heating state of the heating element was 9). Then a total of 10 g of butter (Belolive^^®^^) was melted for another two minutes at state 7 until skim disappeared. One burger per experiment was put in the pan and fried at heating state 5 for 4.5 min for each side (total cooking time 9 min). The fried burger was lifted out of the pan and cooled down for 10 min on a plate followed by determination of the weight. During the process of pan-frying, geometric center and surface temperatures (both top and bottom surface) of three additional burgers were monitored and recorded with a data logger (Testo 177-T4). The thermocouples were bent and inserted at ca. 3 mm depth in the burger so that they could measure temperature in a relatively small top/bottom surface layer of the burger. This temperature is henceforward called burger surface temperature. As a side-remark, it should be noted that the surface of a pork meat burger is not a flat and smooth surface and temperature of the (sub) surface of the burger may be very location specific. As soon as the burger was turned, the probes were immediately put back in. To measure the core temperature, a wireless temperature logger (DS1922T iButton, Maxim Integrated Products, Sunnyvale, CA, USA) was placed into the center of the burger. The burger core temperature profile was used to calculated the process lethality (*F*-value) using an Excel spreadsheet^2^ based on the formula below

$$F=\int_{0}^{t}10^{(T-T_{ref})/z}\text{d}t$$

where *T* is the core temperature (°C) at a time *t* (min) and *T*_ref_ is a reference temperature (60°C was used in this study). According to a previous study (Murphy et al., 2006), in ground pork the *z* value is 5.89°C for *Salmonella* and 5.92°C for *L. monocytogenes*.

A representative 10 g sample, a strip of ca. 1 cm wide from the middle of the fried burger, was taken for microbial analysis. Enumeration of *S. enterica* or *L. monocytogenes*, TPC, total coliforms and LAB was performed as described above. For the samples where no surviving *S. enterica* or *L. monocytogenes* were found by enumeration, duplicate 25 g samples were used to test a complete inactivation of pathogens by the enrichment method. The enrichment of *S. enterica* and *L. monocytogenes* was carried out as previously described by Siro et al. (2006). For *S. enterica*, a 25 g sample was blended with 225 ml of Buffered Peptone Water (BPW, Oxoid) and incubated at 37°C for 24 h. From the primary enrichment, 0.1 ml of the aliquot was transferred into 10 ml of Rappaport-Vassiliadis broth (RVS, Oxoid) and incubated at 42°C for a further 24 h before plating out on XLD plates. For *L. monocytogenes*, the primary enrichment was done in Demi-fraser enrichment broth (Oxiod) at 30°C for 24 h. Then a 0.1-ml of the primary enrichment broth was subcultured into the secondary enrichment broth (10 ml of Fraser) and incubated at 37°C for 24 h. Afterward samples were streaked onto ALOA plates.

### Data Analysis

Growth and inactivation studies for both pathogens were performed in triplicates. The mean of the duplicated plate counts per repetition was determined and converted to log_10_ values, and plotted versus time. Growth curves were fitted with “DMFit online^3^” using the Baranyi and Roberts (1994). Cell counts below the detection limit of 5 CFU/g were excluded in the calculation of curves, but indicated as separate data points on *x*-axis in the same figure. The growth parameters including lag time (*λ*), maximum growth rate (μ_max_), and maximum population density (*y*_max_) were determined. Inactivation data were analyzed by linear and non-linear models by the software GInaFiT (version 1.6) (Geeraerd et al., 2005). The goodness of fit of the models was assessed using adjusted regression coefficient ($R_{adj}^{2}$). The kinetic parameters from the best fit model were reported. Statistical interpretation of differences among parameters was determined using ANOVA analysis (SPSS statistical software, Inc., Chicago), using 95% confidence limits.

## Results

### Growth of *S. enterica* and *L. monocytogenes* in Broth

Growth curves of a cocktail of three strains of *S. enterica* or *L. monocytogenes* in broth exhibited a classical sigmoidal behavior (not shown). Variation among replications was found to be not significant (*P* > 0.05), and thus the growth data were averaged. At both initial densities, the maximum growth rate of *S. enterica* and *L. monocytogenes* was estimated to be about 0.021 and 0.066 log_10_ CFU/ml/h, respectively. Due to the longer lag time (ca. 60 vs. 17 h) and lower growth rate, the time needed to reach stationary phase for *S. enterica* was more than double that of *L. monocytogenes*.

### Growth of *S. enterica* and *L. monocytogenes* in Ground Pork with a Natural Microbiota

**FIGURE 2:**
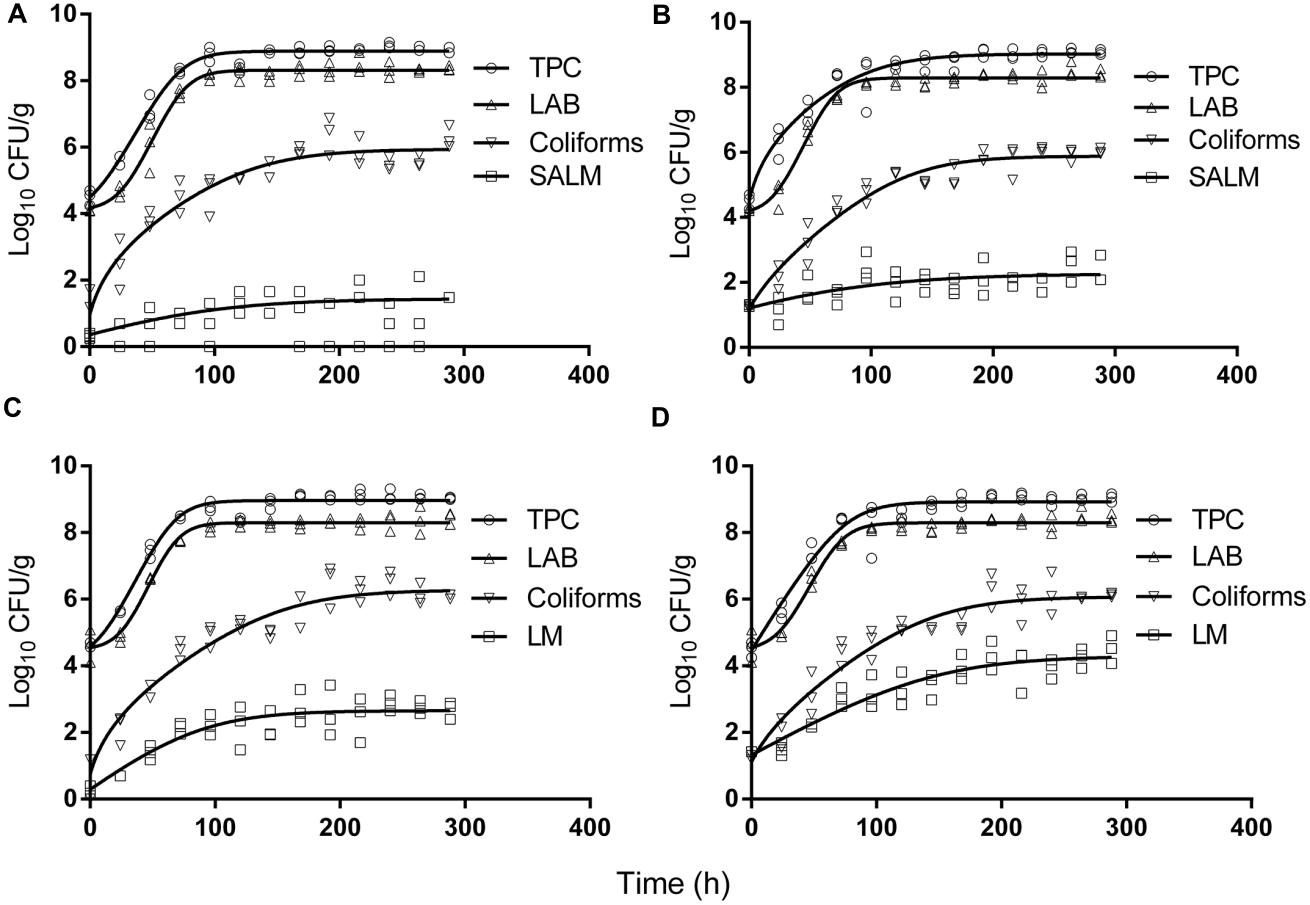
**Growth of indigenous microbiota and *S. enterica* (SALM) (A,B) or *L. monocytogenes* (LM) (C,D) at low initial densities (A,C ∼1 CFU/g; B,D ∼10 CFU/g) at 10°C in ground pork meat.** Solid lines are regression lines fitted with Baranyi and Roberts (1994) model.

The initial concentration of TPC, LAB, and coliforms in the ground pork were ca. 4.6, 4.4, and 1.5 log CFU/g, respectively, which indicated satisfactory initial microbial quality of the ground pork meat. Growth curves of TPC, coliforms, and LAB with different inoculum levels of *S. enterica* or *L. monocytogenes* at 10°C are presented in **Figure 2**. After ca. 4 to 5 days all the indigenous bacteria reached the stationary phase of growth. TPC reached its maximum value of ca. 8.9 log CFU/g, LAB at 8.3, whereas 5.9 log CFU/g for coliforms. The maximum growth rates of the indigenous bacteria were similar to each other (*P* > 0.05) regardless of their initial levels or inoculated pathogens (**Tables 1** and **2**).

**Table 1:** Growth parameters of indigenous microbiota (TPC, total plate count; LAB, lactic acid bacteria) and *S. enterica* (SALM) in ground pork meat at 10°C determined by Baranyi and Roberts (1994) model.

Initial density of SALM (log CFU/g)	Growth parameter	Bacteria count (log CFU/g)			
		SALM	TPC	LAB	Coliforms
0.33 ± 0.08	*y*_0_ (log CFU/g)	0.45 ± 0.14	4.42 ± 0.16	4.17 ± 0.10	1.81 ± 0.31
	*y*_max_ (log CFU/g)	1.39 ± 0.08	8.88 ± 0.06	8.31 ± 0.04	5.84 ± 0.16
	*μ*_max_ (h^-1^)	0.007 ± 0.002	0.055 ± 0.004	0.067 ± 0.004	0.031 ± 0.005
	*λ* (h)	N/A^a^	N/A	19.9 ± 3.2	N/A
1.30 ± 0.04	*y*_0_ (log CFU/g)	1.14 ± 0.41	4.70 ± 0.24	4.21 ± 0.17	1.27 ± 0.28
	*y*_max_ (log CFU/g)	2.13 ± 0.10	8.88 ± 0.09	8.29 ± 0.06	5.69 ± 0.13
	*μ*_max_ (h^-1^)	0.010 ± 0.005	0.054 ± 0.006	0.068 ± 0.008	0.038 ± 0.004
	*λ* (h)	N/A	N/A	16.2 ± 5.0	N/A

*^a^Not applicable.*

**Table 2:** Growth parameters of indigenous microbiota (TPC, LAB) and *L. monocytogenes* (LM) in ground pork meat at 10°C determined by Baranyi and Roberts (1994) model.

Initial density of LM (log CFU/g)	Growth parameter	Bacteria count (log CFU/g)			
		LM	TPC	LAB	Coliforms
0.31 ± 0.12	*y*_0_ (log CFU/g)	0.27 ± 0.19	4.49 ± 0.21	4.56 ± 0.12	1.46 ± 0.34
	*y*_max_ (log CFU/g)	2.56 ± 0.08	8.95 ± 0.08	8.30 ± 0.04	6.07 ± 0.19
	*μ*_max_ (log CFU/g/h)	0.023 ± 0.004	0.058 ± 0.006	0.067 ± 0.001	0.034 ± 0.005
	*λ* (h)	N/A^a^	N/A	19.9 ± 3.7	N/A
1.43 ± 0.01	*y*_0_ (log CFU/g)	1.43 ± 0.18	4.63 ± 0.12	4.48 ± 0.15	1.38 ± 0.29
	*y*_max_ (log CFU/g)	4.18 ± 0.13	8.79 ± 0.08	8.29 ± 0.05	5.88 ± 0.16
	*μ*_max_ (log CFU/g/h)	0.016 ± 0.002	0.060 ± 0.007	0.061 ± 0.006	0.034 ± 0.004
	*λ* (h)	N/A	N/A	16.2 ± 5.0	N/A

*^a^Not applicable.*

*Salmonella enterica* cells were able to multiply at both inoculum levels. However, the population increased by less than one log unit only, even after enforced long time (12 days) storage at this abusive temperature of 10°C. Increase of *S. enterica* starting from ca. 20 CFU/g occurred with limited variation (SD < 0.5 log CFU/g, **Figure 2B**) compared with the samples starting from a few (ca. 2) CFU/g which ranged from <0.7 (detection limit) to 2.1 log CFU/g (**Figure 2A**). Under the same enforced abusive storage conditions *L. monocytogenes* grew exponentially (**Figures 2C,D**) up to a maximum value of 2.6 and 4.2 log CFU/g, respectively (**Table 2**) after 12 days at 10°C. The increase of *L. monocytogenes* starting from ca. 2 and 27 CFU/g was 2.3 and 2.8 log units, respectively. The variation of the observed values of *L. monocytogenes* among replicates was lower than for *S. enterica*.

### Thermal Inactivation of *S. enterica* and *L. monocytogenes* in Broth

**FIGURE 3:**
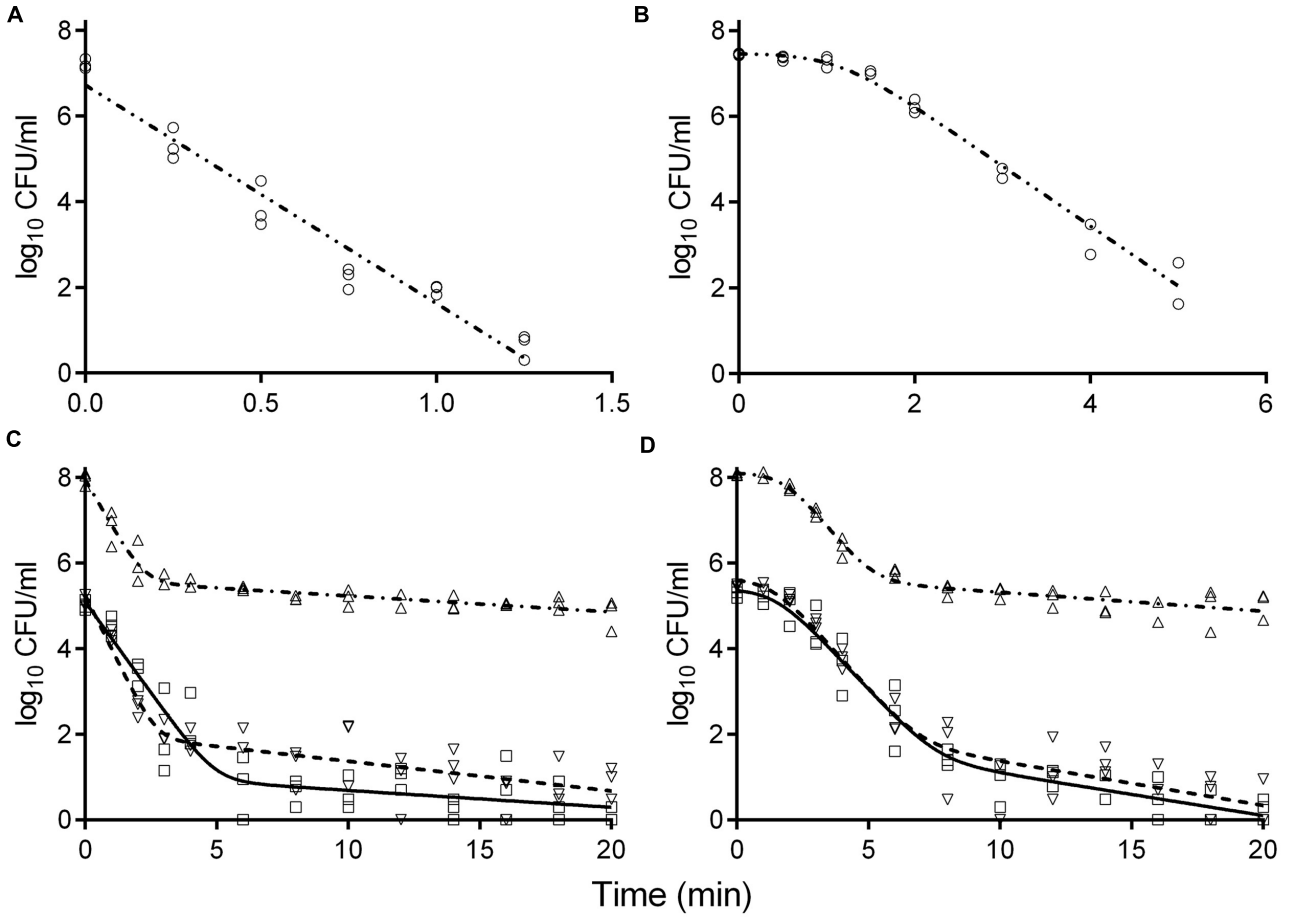
**Inactivation curves of *S. enterica* LFMFP 872 (A,C) and *L. monocytogenes* LFMFP 392 (B,D) at 60°C with different challenge methods (reference method (∘), model (-⋅⋅-); method II (Δ), model (-⋅-)) and broth (standard BHI (∇), model (- - - -); adjusted BHI (□), model (—))**.

Survival curves of *S. enterica* and *L. monocytogenes* strains obtained by the reference method are shown in **Figures 3A,B**. The *S. enterica* curves were fitted by the log-linear model. For all regressions, the $R_{adj}^{2}$ values were larger than 0.95 (data not shown). Decimal reduction time or *D* values were determined from the maximum inactivation rate (*k*_max_, *D* value = ln(10)/*k*_max_). *D* values of *S. enterica* strains ranged from 0.20 to 0.24 min (**Table 3**). Shoulders were observed on all inactivation curves of *L. monocytogenes* and were fitted to a log linear model with a shoulder (Geeraerd et al., 2000). The fittings yielded $R_{adj}^{2}$ values from 0.97 to 0.99. The shoulder length (*S*_l_) ranged from 0.52 to 1.13 min. *D* values of *L. monocytogenes* were more than twice higher than those of *S. enterica*. In general a minimum process of 6*D* reductions in the numbers of target microorganisms is recommended for pasteurized foods (International Commission on Microbiological Specifications of Foods [ICMSF], 2005; FDA, 2011a). The *t*_6D_ values, expressing the time needed to obtain six decimal reductions (Buchanan et al., 1993) of *S. enterica* and *L. monocytogenes* are given in **Table 3**. Since shoulders were observed on *L. monocytogenes* inactivation curves, *t*_6D_ of *L. monocytogenes* strains are larger than six times the *D* values.

**Table 3:** Impact of heating procedure and challenge broth on the thermal resistance (*D* values ± *SD*) of SALM and LM heated at 60°C.

Bacterial strain		Reference method (method I)		Apparent *D* value from method II using standard BHI and from high concentration (min)^a^	Apparent *D* values from method II using standard BHI and from low concentration (min)^a^	Apparent *D* values from method II using adjusted BHI and from low concentration (min)^a^	*D* values from published data (min)
		*D* value (min)	*t*_6*D*_(min)				
SALM	872	0.20 ± 0.02	1.18 ± 0.11	0.53 ± 0.08	0.59 ± 0.13	0.72 ± 0.39	0.75 ± 0.74
	875	0.22 ± 0.01	1.33 ± 0.04	0.77 ± 0.11	0.64 ± 0.02	0.95 ± 0.59	(*n*^b^ = 62)
	877	0.24 ± 0.01	1.46 ± 0.06	0.93 ± 0.10	0.62 ± 0.08	1.42 ± 0.44	
LM	392	0.72 ± 0.03	5.45 ± 0.42	1.29 ± 0.22	1.37 ± 0.46	1.26 ± 0.25	1.32 ± 0.84
	421	0.68 ± 0.02	4.65 ± 0.10	1.01 ± 0.26	1.05 ± 0.10	1.07 ± 0.31	(*n*^b^ = 61)
	491	0.71 ± 0.04	5.40 ± 0.46	1.44 ± 0.35	1.42 ± 0.40	1.90 ± 0.50	


*^a^D values were calculated from the slope of the initial linear portion of each survival curve.*

*^b^*n*, Number of *D* values reported from ComBase database and literature*.

The inactivation curves obtained by the method II of thermal treatment (inoculated via the inner wall in the tube instead of immediately in the suspension) showed a biphasic shape. Typical curves are shown in **Figures 3C,D**. Survivor curves showed initially 2 to 3 log reductions, followed by prolonged tailing in which the numbers only slightly decreased further. A zero point was not achieved even after 20-min thermal challenge at 60°C. It deserves attention that the apparent *D* values, which were calculated from the initial log-linear part of the biphasic curves obtained by method II, were 1.5- to 2.9-fold larger than those obtained using the reference method I (**Table 3**). This is important to be noticed as the exact laboratory procedure to determine *D* values is not always described in detail in scientific literature and this highlights the fact that small deviations in elaborating the laboratory procedure for *D* values determination may impact the outcome.

When the pathogen cells were thermally treated at an initial concentration of ca. 10^5^ CFU/ml, inactivation curves showed the same pattern as the high initial concentration (ca. 10^8^ CFU/ml) (**Figures 3C,D**). The apparent *D* values were more or less invariable (**Table 3**). Apparent *D* values of each strain thermally treated in standard and adjusted BHI are also listed in **Table 3**. The strains treated in adjusted BHI (pH 5.6, 1.5% NaCl) showed higher apparent *D* values than those in standard BHI, especially for *S. enterica*.

### Inactivation of *S. enterica* and *L. monocytogenes* in Pork Meat Burger by Simulated Home Pan-frying

**FIGURE 4:**
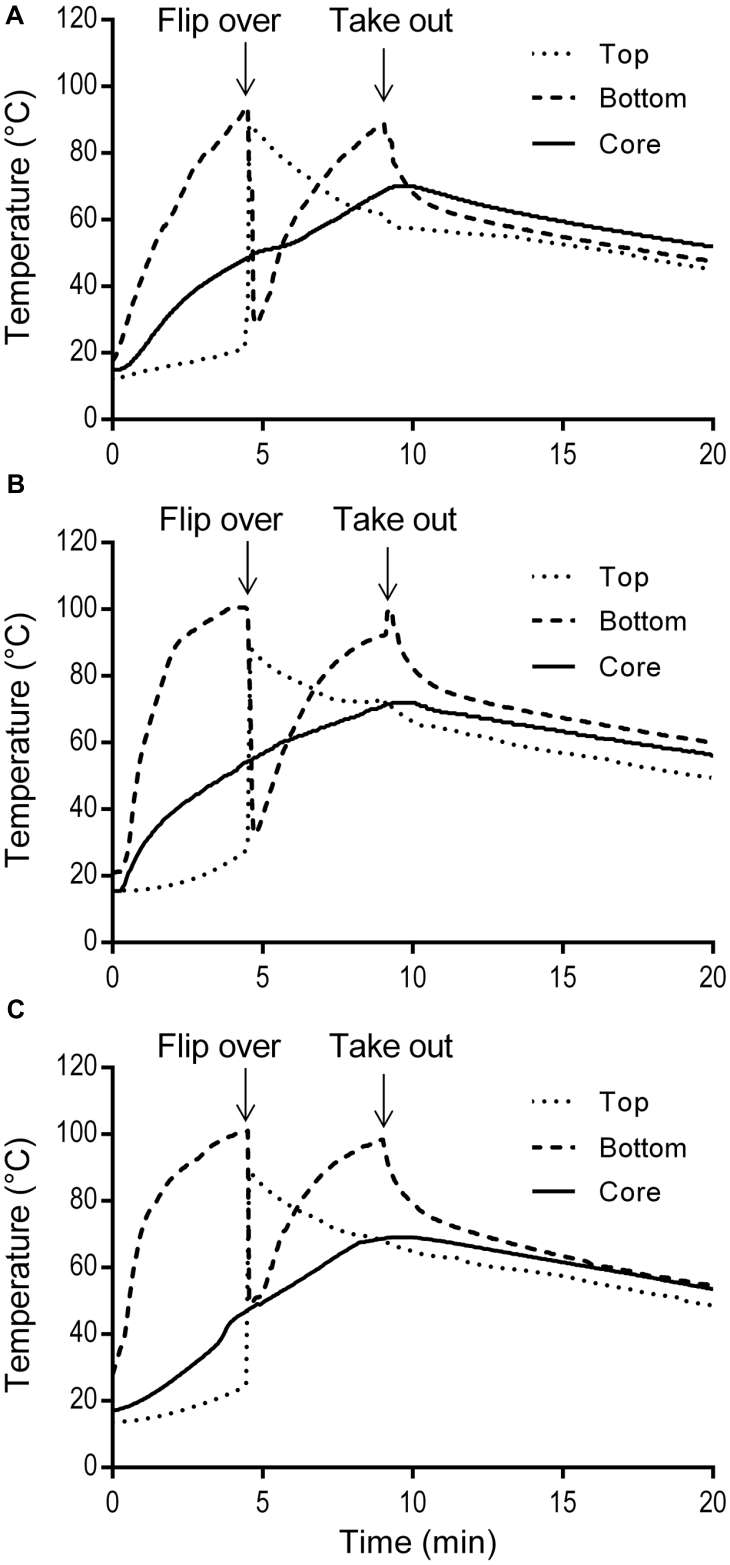
**Temperature profiles of three replicate pork meat burgers (A,B,C) during simulated home pan-frying**.

The simulated home pan-frying procedure used in this study resulted in 30.4 ± 1.7% weight loss of the burgers. It was similar as a standard pan-frying procedure applied by Danowska-Oziewicz (2009) where the cooking loss was 28%. The temperature profiles of three burgers during pan-frying and cool-down at ambient temperature on the serving plate are presented in **Figure 4**. The temperatures of the burgers bottom rose sharply to the maximum (93.9–100.6°C) before flipping, while the increase on the top was very limited. The bottom temperatures were higher than the core temperatures, and this difference increased with time. After flipping, the (new) bottom temperature increased quickly while the (new) top temperature decreased gradually. During cooling down on the serving plate the bottom temperatures of the burgers immediately started to decrease exponentially, while the core temperature still slightly increased due to heat conduction. The peaks of the core temperatures, which ranged from 69.0 to 71.9°C, were reached at ca. 0.3 min after taking the pork meat burgers out from the pan.

To evaluate the efficacy of thermal treatment during this simulated home pan-frying on the inactivation of pathogens in the meat, *F* values were calculated in pork burger as the equivalent time needed to reduce *S. enterica* or *L. monocytogenes* at 60°C. *F* values were obtained according to the core temperature profiles of the burgers (**Figure 4**). The calculated *F* values for *S. enterica* were 115, 282, and 123 min for three replicates, respectively; and for *L. monocytogenes* 113, 276, and 121 min. All the *F* values were obviously much higher than the expected time needed for 6 log reductions of both pathogens. After the pan-frying procedure pathogens are thus expected to reduce to undetectable levels as in the present study the initial contamination levels of *S. enterica* and *L. monocytogenes* in pork meat burgers (after prior storage for 5 days at 10°C) were ca. 1.95 log CFU/g and 3.10 log CFU/g, respectively (**Table 4**). As expected no *S. enterica* were recovered from all the samples after enrichment in 25 g of pan-fried pork meat burger. Accordingly, at least a 3.3-log unit reduction of *S. enterica* was obtained. However, the presence of *L. monocytogenes* was detected in three out of six of the 25 g pan-fried pork burger samples, so 2.4- to 4.5-log units reduction was achieved in these three burgers, but no 6-log unit reduction was obtained. As for the indigenous microbiota the number of surviving bacteria was significantly reduced. The mean reductions of TPC and LAB were all over 6 log units. Regarding coliforms, this microbial group was, in all cases below the detection limit of 5 CFU/g.

**Table 4:** Effects of pan frying on the inactivation of SALM or LM and indigenous microbiota count (TPC, LAB) in pork meat burgers.

Bacteria	Initial density (log CFU/g)	Final density^a^ (log CFU/g)	After pan-frying (log CFU/g)	Detection per 25 g^b^
**Pork meat burgers inoculated with *S. enterica***				
SALM	1.28 ± 0.04	1.95 ± 0.07	<0.7	0/6
TPC	4.59 ± 0.23	8.64 ± 0.13	1.72 ± 0.64	N/A^c^
LAB	4.63 ± 0.49	7.99 ± 0.42	1.38 ± 0.25	N/A
Coliforms	1.53 ± 0.09	5.19 ± 0.13	<0.7	N/A
**Pork meat burgers inoculated with *L. monocytogenes***				
LM	1.43 ± 0.01	3.10 ± 0.2	<0.7	3/6^d^
TPC	4.31 ± 0.18	8.15 ± 0.51	1.23 ± 0.21	N/A
LAB	3.95 ± 0.24	7.53 ± 0.35	0.80 ± 0.17^e^	N/A
Coliforms	1.23 ± 0.06	4.83 ± 0.18	<0.7	N/A

*^a^Density after storage at 10°C for 5 days.*

*^b^These results present the number of replicates the pathogen was detected in 25 g after enrichment.*

*^c^Not applicable.*

*^d^One of the duplicate samples in each trial was positive.*

*^e^One of the three trials was below the detection limit.*

## Discussion

In this work, we studied the growth of *S. enterica* and *L. monocytogenes* in artificially contaminated ground pork meat during storage under reasonably foreseen temperature abuse at 10°C. Subsequently, the inactivation of these pathogens – which were allowed to grow for 5 days at 10°C in the pork burger – was determined using a pan-frying procedure routinely practiced in Belgian domestic settings. The growth and inactivation results in the pork meat burgers were compared with those obtained in laboratory media such as BHI broth.

The survival and growth of *S. enterica* and *L. monocytogenes* in ground pork meat was monitored for up to 12 days of storage at 10°C. It is obvious that the meat was spoiled as of day 5: the TPC reached maximum levels. Monitoring of pathogens’ behavior was continued to assess whether there was still outgrowth or rather survival or die-off of *S. enterica* and *L. monocytogenes* in presence of competition with these maximum levels of indigenous microbiota and their metabolites. Also this enabled maximum comparison between behavior in the meat versus BHI broth and predictions obtained by the mathematical models. The growth parameters of *S. enterica* and *L. monocytogenes* in BHI were generally in agreement with previous selected reports from Combase database and literature when selecting experimental conditions comparable to those in the present study (culture media of pH 7–7.5, aw 0.99–1.00, incubated at 10°C). The Combase reported growth rates of *S. enterica* in broth at 10°C varied from 0.020 to 0.030 log CFU/ml/h, with an average of 0.028 (4 reported values). As for *L. monocytogenes*, the growth rates ranged from 0.041 to 0.082 log CFU/ml/h with an average of 0.054 (21 reported values). In our study, at both initial densities, the maximum growth rate and *y*_max_ of *S. enterica* or *L. monocytogenes* was estimated to be similar. Thus results in the present study agreed with previous reports where the growth of pathogens in sterile broth was usually independent of initial density and *y*_max_ is usually not greatly affected by growth conditions (Buchanan and Klawitter, 1991).

As shown, both *S. enterica* and *L. monocytogenes* have the ability to multiply in ground pork at 10°C in the presence of a substantial numbers of indigenous microbiota. Still, it was observed that the growth of pathogens ceased when the indigenous microbiota reached its maximum population density. This is probably due to microbial competition between pathogens and the indigenous microbiota. This phenomenon has been referred to as the “Jameson effect” (Jameson, 1962). It is noted that for both *S. enterica* and *L. monocytogenes* in the pork meat, *y*_max_ was dependent on the initial dose; *y*_max_ was higher at higher initial pathogen contamination level, which is inconsistent with the results obtained in BHI broth. The difference in *y*_max_ could also be attributed to the Jameson effect by the indigenous microbiota in ground pork meat. A number of studies have been done on the growth of pathogens in sterilized ground meat where no competition occurred. Velugoti et al. (2011) studied the growth of *Salmonella* sp. in sterile ground pork meat. At 10°C, *S. enterica* reached a maximum population of 8.3 log CFU/g with a maximum rate of 0.018 log CFU/g/h, both of which were much higher than those values obtained in the present study. Mbandi and Shelef (2001) investigated the growth of *S. enterica* and *L. monocytogenes* in sterile ground beef at 10°C: numbers of both pathogens increased from 3.5 to approximately 8.0 log CFU/g after 20 days of storage. Indigenous microbiota in raw ground meat are thought to consist of a variety of microorganisms that can inhibit the growth of pathogens. Ingham et al. (2007) studied the growth of pathogens in meat with relatively low levels of indigenous biota (≤3.5 log CFU/g) and relatively high levels of inoculated pathogens (4.6 log CFU/g). An online software for evaluating the safety of meat was developed based on their study^4^. This online tool predicted for *Salmonella* a growth of 6.6 log units in ground pork after 12-days storage at 10°C. However, we observed only less than one log unit increase of *S. enterica* and ca. 2.5 log units increase of *L. monocytogenes*. Similarly, Oscar (2007) reported that at 10°C, the growth of *S. enterica* from a low initial density in ground chicken with a natural microbiota was also very limited, from 1.1 to 1.8 log MPN or CFU/g.

Thermal inactivation of *Salmonella* and *L. monocytogenes* has been studied extensively resulting in a wide range of *D* values. It is well known that the inactivation dynamics may be influenced by various factors including the bacterial strain of the species, the physiological state of microbial cells, heating and recovery conditions (Smelt and Brul, 2014). Average *D* values of *Salmonella* and *L. monocytogenes* at 60°C as reported in broth or buffers (pH 7–7.5, a_w_ 0.99–1.00) were listed and compared to the ones estimated in the present study (**Table 3**). The average published *D* values for *Salmonella* and *L. monocytogenes* were 0.75 and 1.32 min, respectively. Thus, the *D* values obtained in the BHI broth in the present study were within the same order of magnitude.

For almost one century, the food industry assumed that thermal inactivation followed first-order kinetics during the estimation of the outcome of a thermal treatment on the survival of microorganisms. However, there is growing evidence to support that the inactivation of microbial cells does not always follow the traditional first-order kinetics, especially during a mild thermal treatment (Augustin et al., 1998; Valdramidis et al., 2006). In the present study, shoulders were observed on *L. monocytogenes* survival curves. It has been a consensus that *D* values should be used with care when the isothermal survival curves are not really log-linear (Peleg, 2006). However, in many published articles, no inactivation curves are shown, but only *D* values. It is not clear if the original data were indeed log-linear so that the derived *D* values can have a clear meaning. Therefore, it is recommended that the ‘*D* values’, including the ones reported in literature are critically assessed. The *t*_xD_, an alternative concept for thermal microbial inactivation, was developed to describe microbial heat resistance (Buchanan et al., 1993). It describes the time *t* required for *x* log units reductions in the microbial population. In this concept, the deviations from the first-order kinetics were taken into account when estimating the effectiveness of a thermal treatment instead of excluding any shoulders and tails. Meanwhile, the use of *t*_xD_ rather than *D* values when communicating the performance of food inactivation processes has been accepted by many researchers (Heldman and Newsome, 2003; Valdramidis et al., 2005).

As established in the present study, the heat resistance may be affected by the heating method. Various methods of thermal treatment have been applied in evaluating heat resistance of bacteria in a laboratory media, e.g., heating in water baths using capillary tubes, test tubes, glass ampoules completely immersed in the water, and heating using pasteurization, submerged-coil heating apparatuses etc. (Sorqvist, 2003). The test tube method is one of the commonly used due to the advantage of easy handling. The two thermal treatment methods applied in our study produced different patterns of inactivation curves and *D* values. Similar observations for bacterial cells or mold spores have been reported in previous studies when the test organism was heated in incompletely submerged capped tubes (Schuman et al., 1997; Zimmermann et al., 2013). The cells coating the walls above the level of the water bath were regarded to be responsible for this tailing phenomenon; these cells were not exposed to the intended temperature of inactivation. The pathogens’ strains also showed higher heat resistance in broth with pH adjusted to 5.6 and an increased (1.5%) NaCl concentration. The effect of the pH on the heat resistance was similar to that observed previously (Blackburn et al., 1997; Mañas et al., 2003; Arroyo et al., 2009). There was an optimum pH for survival of cells, increasing acidity or alkalinity increased the rate of inactivation. It has been reported that maximum heat resistance of several pathogens is obtained at slightly acidified media (Blackburn et al., 1997). Furthermore, 1.5% NaCl in adjusted BHI had a heat protective effect. Based on the above, it needs to be recognized that the thermal inactivation kinetics of bacterial pathogens can be affected by the test procedures and types of challenge media. It is important to use suitable methodology in assessing the thermal resistance and clearly state the test conditions.

Based on the *6D* values of each three strains obtained in BHI broth in this study, 1.5 and 5.5 min thermal treatment at 60°C are deemed to be sufficient to achieve a 6-log unit reduction for *S. enterica* and *L. monocytogenes*, respectively. However, considering the increased heat resistance of pathogens in a food matrix versus laboratory media (Kenney and Beuchat, 2004), longer time may be needed in meat burgers to get 6 log units reductions for pathogens. O’Bryan et al. (2006) summarized the thermal resistance of *S. enterica* and *L. monocytogenes* in meat and poultry and great variation was shown. At 60°C, *D* values of *S. enterica* varied from 3.83 to 8.5 min and *L. monocytogenes* varied from 0.31 to 16.7 min. Even when the highest *D* values were used for the worst-case scenario considered, the pan-frying process should be sufficient to result in a 6 log reduction of both pathogens based on the calculated *F* values. However, the presence of *L. monocytogenes* in 25 g was detected in three out six of the pan-fried pork meat burgers samples. This result may be explained by several facts. Firstly, the pathogens in the pork burgers in this study were inoculated at a low level (ca. 10^2^ CFU/g) and grew at 10°C for 5 days on the meat particles. It has already been reported that food type and composition (e.g., percentage fat) may have a protective effect on thermal inactivation. For example, Murphy et al. (2000) observed increased *D* values for a mixture of six *Salmonella* serotypes and *Listeria innocua* M1 when comparing the inactivation in chicken breast meat patties and a peptone agar aqueous solution. Secondly, the bacteria were constrained to grow as colonies. In Velliou et al. (2013) it was shown how *E. coli* K12 and *Salmonella* Typhimurium, grown as colonies of various sizes in a matrix gelled with xanthan gum, display a higher thermal resistance when compared with planktonic cells. The surviving *L. monocytogenes* after pan-frying may be a potential risk for food safety. Nevertheless, it is supposed that a concentration of *L. monocytogenes* not exceeding 100 CFU/g of food at the time of consumption poses limited risk to the consumers (Nørrung, 2000).

Based on the growth and inactivation results in ground pork meat as obtained in the present study, it was established that *L. monocytogenes* grow faster and reaches a higher population density, and there were survivors after a simulated home-frying procedure. As such, it can be inferred that a thermal process that ensures destruction of *L. monocytogenes* in ground pork will also provide an adequate reduction of natural microbiota and other less heat resistant pathogens such as *Salmonella* possibly present in the pork meat burger. This coincides with previous recommendations that *L. monocytogenes* can be considered as the target organism for thermal inactivation (Rocourt et al., 2000; International Life Sciences Institute [ILSI], 2012).

## Conclusion

Results of this study in particular demonstrated that growth and thermal inactivation data based on laboratory experiments executed in broths show a clear difference with that of what can be expected in actual food. In the present study, both an overestimation of the extent of growth and an overestimation of the extent of inactivation was noticed. The former overestimation leads to a fail-safe situation, however, the latter overestimation is a fail-dangerous outcome. When applying outcomes from models based on laboratory media and condition to foods it is thus important to validate these models carefully and take into account differences that might occur due to other composition, texture and physico-chemical characteristics of the food matrix and indigenous competing microbiota, described as different types of *errors* in Pin et al. (1999) and Miconnet et al. (2005). In the present study the *intermediate error* includes the competition with the natural microbiota occurring at realistic levels of pathogen contamination. The *overall error*, related with the difference between naturally occurring and artificially contaminating pathogens, remains to be investigated for ground pork meat naturally contaminated with *S. enterica* or *L. monocytogenes*.

## Conflict of Interest Statement

The authors declare that the research was conducted in the absence of any commercial or financial relationships that could be construed as a potential conflict of interest.

## Footnotes

1 http://www.combase.cc

2 http://www.namif.org/content/process-lethality-spreadsheet

3 https://browser.combase.cc/DMFit.aspx

4 http://www.meathaccp.wisc.edu/therm
